# Previous Hamstring Injury Does Not Impact Biceps Femoris Long Head Fascicle Length and Eccentric Knee Flexion Strength Changes Following a Low‐Volume Resistance Training Intervention in Elite Under‐20‐Year‐Old Male Gaelic Footballers

**DOI:** 10.1002/ejsc.70204

**Published:** 2026-06-25

**Authors:** Tommy Mooney, Kevin Cronin, Shane Malone, Neil Welch, Kieran Collins, Jack Hickey

**Affiliations:** ^1^ Gaelic Sports Research Centre Technological University Dublin Dublin Ireland; ^2^ Sports Medicine Research Department UPMC Sports Surgery Clinic Dublin Ireland; ^3^ Diagnostic Imaging Department, School of Medicine University College Dublin Dublin Ireland; ^4^ Department of Sport Science and Nutrition Maynooth University Maynooth County Kildare Ireland; ^5^ Sports Performance, Recovery, Injury and New Technologies (SPRINT) Research Centre Australian Catholic University Melbourne Australia

**Keywords:** muscle architecture, nordic hamstring exercise, Romanian deadlift, sprinting, ultrasound

## Abstract

This study reports biceps femoris long head (BFlh) fascicle length and eccentric knee flexion strength changes following a 9‐week in‐season low‐volume resistance training intervention in elite under‐20‐year‐old (U20) male Gaelic footballers with and without previous hamstring injury. We included 26 male Gaelic footballers (age = 18.5 ± 0.5 years; height = 183.1 ± 5.1 cm; body mass = 79.2 ± 7.9 kg) in this study, with 10 of these participants reporting previous hamstring injuries within the last 12 months. Participants performed two sets of four Nordic hamstring exercise (NHE) repetitions and three sets of six staggered‐stance Romanian deadlift (RDL) repetitions once per week for nine weeks. Before and after the intervention, BFlh fascicle length was assessed via ultrasound and eccentric knee flexion strength was measured during the NHE. We ran separate linear mixed‐effects models for BFlh fascicle length and eccentric knee flexion strength, with fixed‐effects of time (baseline and follow‐up), limb‐type (previously injured and contralateral uninjured limbs of previously injured participants and between‐limb average of uninjured participants) and the time*limb‐type interaction. The linear mixed‐effects model for BFlh fascicle length revealed a significant main effect of time (*β* = 0.31 cm; SE = 0.15 cm; *p* = 0.04), while the time*limb‐type interaction was not statistically significant. The linear mixed‐effects model for eccentric knee flexion strength revealed no significant increase in eccentric knee flexion strength (*β* = 9.53 N, SE = 12.97 N, *p* = 0.47), while the time*limb‐type interaction was not statistically significant. These findings suggest changes to BFlh fascicle length and eccentric knee flexion strength from baseline to follow‐up were comparable across all limb‐types.

## Highlights

- The Nordic hamstring exercise and staggered‐stance Romanian deadlift can be implemented as a weekly resistance training intervention in an elite under‐20‐year‐old male Gaelic football team during the first nine weeks of the competitive season.
- Following this intervention, biceps femoris long head fascicle length significantly increased, while eccentric knee flexion strength did not change significantly.
- Previous hamstring injury did not alter changes to biceps femoris long head fascicle length or eccentric knee flexion strength.

## 1 Introduction

Hamstring injuries remain a common burden across different field‐based team sports played around the world (Maniar et al. 2023). The most common risk factor for hamstring injury in these sports is a previous hamstring injury (Green et al. 2020), and this cannot be modified. However, a previously injured athlete's risk of hamstring injury can be mitigated by their biceps femoris long head (BFlh) fascicle length and eccentric knee flexion strength (Opar et al. 2022; Timmins et al. 2016). These modifiable variables are being increasingly monitored in field‐based team sports to try to reduce hamstring injury risk, especially for previously injured athletes.

In elite Australian football, BFlh fascicle length and eccentric knee flexion strength have both been shown to improve during pre‐season and early in‐season periods, but only in uninjured athletes, not those with a previous hamstring injury (Opar et al. 2015; Timmins et al. 2017). These findings partly support the hypothesis that previous hamstring injury limits BFlh fascicle length and eccentric knee flexion strength adaptations to training (Fyfe et al. 2013). However, this hypothesis is only partly supported, as exposure to training interventions that improve BFlh fascicle length and eccentric knee flexion strength were not standardised or controlled for in these studies (Opar et al. 2015; Timmins et al. 2017).

The Nordic hamstring exercise (NHE) and Romanian deadlift (RDL) are both effective resistance training interventions for increasing BFlh fascicle length and eccentric knee flexion strength (Behan et al. 2022; Crawford et al. 2025; Lacome et al. 2020; Medeiros et al. 2020; Presland et al. 2018). These evidence‐based interventions tend to involve training frequencies of at least twice per week, which can be challenging to implement around on‐field training and matches in field‐based team sports, due to associated fatigue and delayed onset muscle soreness (Behan et al. 2023; Cuthbert et al. 2020). This challenge is made more difficult in Gaelic football, where even at the elite‐level of competition, athletes are strictly amateur, which limits time for resistance training. In this unique context of Gaelic football, where most teams can only implement a standardised resistance training session once per week during the season, it is worth investigating if BFlh fascicle length and eccentric knee flexion strength can be improved following weekly exposure to the NHE and RDL across athletes with and without a previous hamstring injury.

The aim of this study was to investigate if previous hamstring injury impacts BFlh fascicle length and eccentric knee flexion strength changes following a low‐volume in‐season resistance training intervention in an elite under‐20‐year‐old (U20) male Gaelic football team.

## 2 Materials and Methods

### 2.1 Study Design and Participants

To address our research aim, we designed this prospective cohort study with repeated measures, which was approved by the TU Dublin Research Ethics Committee. We invited outfield players from an elite U20 male Gaelic football team to participate in our study. Players were not eligible to participate if they were aged under 18 years, had any current lower limb injury, or played in the goalkeeper position, due to their relatively limited risk of hamstring injury. From the overall squad of 36 players, 26 eligible players (age = 18.5 ± 0.5 years; height = 183.1 ± 5.1 cm; body mass = 79.2 ± 7.9 kg), provided their informed written consent to participate in this study. After providing their consent to participate, each participant self‐reported their history of hamstring injury within the last 12 months by completing Part A of the valid and reliable Hamstring Outcome Score questionnaire (Engebretsen et al. 2008; Tsutsumi et al. 2024; van de Hoef et al. 2021). Of the 26 participants included in this study, 10 participants reported previous hamstring injuries within the last 12 months. Because 3 of these participants reported a bilateral history of hamstring injury within the last 12 months, the 10 previously injured participants included 13 previously injured limbs and 7 contralateral uninjured limbs.

### 2.2 Resistance Training Intervention

**FIGURE 1:**
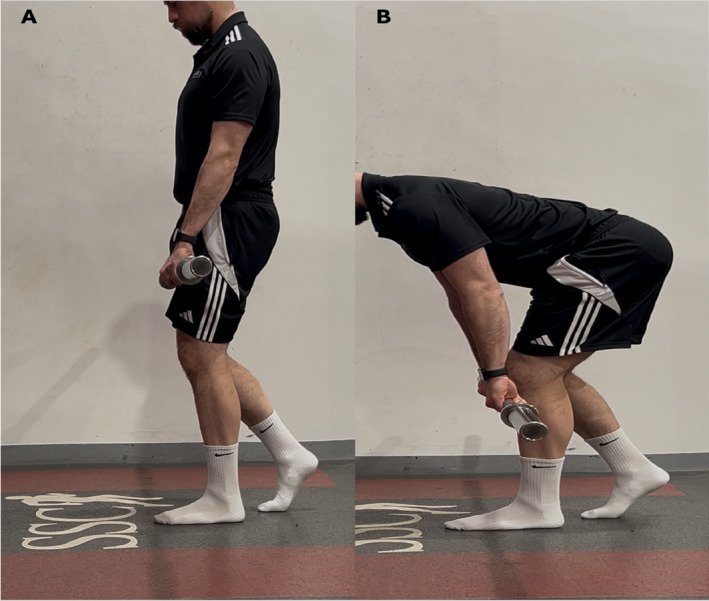
Staggered‐stance RDL technique. Start/finish(A) and mid (B) positions of the Staggered‐stance RDL. Subjects were instructed to place the tip‐top of the contralateral foot in line with the heel and then push hips back under before returning to the start position. RDL, Romanian deadlift.

During the first nine weeks of the 2023/2024 season, resistance training sessions were implemented once per week across the entire squad of athletes, in addition to their typical on‐field training twice per week, and competitive matches once per week. During each resistance training session, participants performed two sets of four NHE repetitions and three sets of six staggered‐stance RDL repetitions under the supervision an Accredited Strength and Conditioning Coach with 10 years of experience. Participants were instructed to only perform the eccentric phase of the NHE, ensuring they lowered their body towards the ground as slowly as possible during each repetition with maximal effort. The staggered‐stance RDL included an eccentric lowering phase and concentric lifting phase (Figure 1), with weight plates added to the barbell in 5 kg increments if a participant's rating of perceived exertion (RPE) was less than 6 on a 0 to 10 RPE scale at the end of a set. We chose to implement the staggered‐stance RDL, as we believed participants with a previous unilateral hamstring injury may have offloaded their previously injured limb if they performed the conventional bilateral‐stance variation of this exercise. Participants completed six staggered‐stance RDL repetitions with each foot in the lead position for each of the three sets prescribed.

### 2.3 Baseline and Follow‐Up Data Collection

Measures of BFlh fascicle length and eccentric knee flexion strength were collected during the week before (baseline) and week after (follow‐up) the 9‐week resistance training intervention. At baseline and follow‐up, both measures were collected on the same day, starting with BFlh fascicle length, then eccentric knee flexion strength, using the methods described below.

#### 2.3.1 Biceps Femoris Long Head Fascicle Length

**FIGURE 2:**
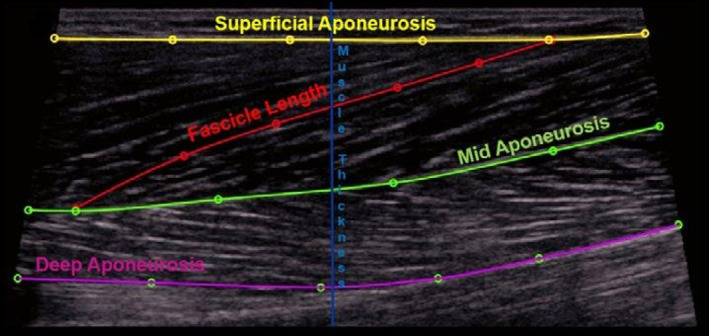
A schematic ultrasound image of bicep femoris long head muscle.

Sonograms of each participant's left and right BFlh muscle were acquired with the participant lying prone on an examination plinth. Coupling gel was placed on the participant's posterior thigh to facilitate transmission of the ultrasound waves intramuscularly. Sonograms were acquired using a Hitachi Noblus ultrasound scanner (Hitachi Medical Systems, UK) with a 92 mm extended‐field‐of‐view (EFOV) transducer (Hitachi EUP‐L53 L), operated by the same experienced radiographer (KC), who has demonstrated excellent test re‐test reliability when measuring BFlh fascicle length using this method (Cronin, Foley, et al. 2022b). This EFOV transducer permits full visibility of an entire fascicle within the BFlh muscle, overcoming limitations associated with using a relatively limited field of view (FOV) transducer, which necessitates the use of extrapolation methods to quantify muscle fascicle lengths (Ritsche et al. 2021; Timmins et al. 2016). These extrapolation methods may be prone to BFlh fascicle length measurement error (Franchi et al. 2020), whereas the EFOV transducer can reliably quantify BFlh fascicle length by permitting full fascicle visibility (Cronin, Delahunt, et al. 2022a; Cronin, Foley, et al. 2022b). Sonograms were analysed using a semi‐automated tracing software tool to measure BFlh fascicle length in centimetres, where a fascicle was clearly illustrated extending from the intermediate aponeurosis to the superficial aponeurosis in the longitudinal plane (Figure 2) (Cronin, Delahunt, et al. 2022a).

#### 2.3.2 Eccentric Knee Flexion Strength

Eccentric knee flexion strength was assessed during the NHE via a portable testing device (Nordbord, Vald Performance, Brisbane, Australia), which has high‐to‐moderate test re‐test reliability (Opar et al. 2013). Following a warm‐up set of three NHE repetitions at 50%, 70% and 90% of maximal effort, respectively, participants performed one set of three maximal effort NHE repetitions, with peak force (N) of each limb recorded during each repetition. Strong verbal encouragement was provided during all testing sessions to ensure participants performed the NHE with maximal effort. Across the three maximal effort NHE repetitions, the average peak force (N) for each limb was calculated and used as the outcome measure of eccentric knee flexion strength.

### 2.4 Statistical Analysis

All statistical analyses were performed using custom written code in the R programing language. We used the ‘lme4’ and ‘lmerTest’ packages to run separate linear mixed‐effects models for each of our dependent variables, BFlh fascicle length and eccentric knee flexion strength. Both models were fit by restricted maximum likelihood, with fixed effects of time (baseline and follow‐up), limb‐type (previously injured and contralateral uninjured limbs of previously injured participants and between‐limb average of uninjured participants) and the time*limb‐type interaction. We used the between‐limb average of uninjured participants in both models because both dependent variables were not significantly different between their left and right lower limbs. Both models included a random intercept of participant ID to account for repeated measures from the same participants. We reported each model's parameter estimates (*β*), standard errors (SE) and *p*‐values, with *p* < 0.05 interpreted as being a statistically significant effect.

## 3 Results

### 3.1 Biceps Femoris Long Head Fascicle Length

**FIGURE 3:**
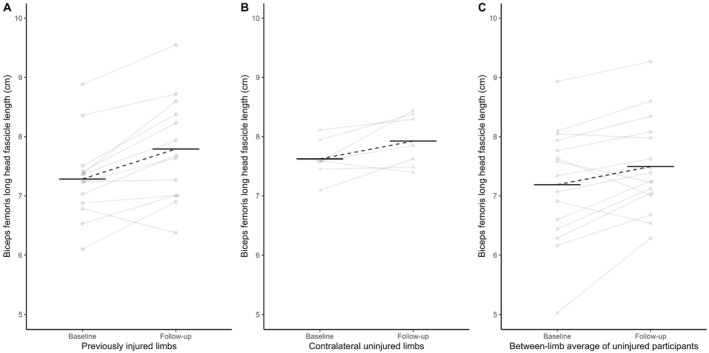
Biceps femoris long head fascicle length (*y*‐axis) at baseline and follow‐up (*x*‐axis) in previously injured limbs (A) and contralateral uninjured limbs (B) of previously injured participants, alongside the between‐limb average of uninjured participants (C). Individual data points and their change over time are shown in grey, while the horizontal black lines and dashed lines represent the average for each limb‐type.

The linear mixed‐effects model for BFlh fascicle length revealed a significant main effect of time (*β* = 0.31 cm; SE = 0.15 cm; *p* = 0.04), indicating an overall statistically significant increase in BFlh fascicle length from baseline to follow‐up. There were no significant baseline differences in BFlh fascicle length between uninjured participants and the previously injured (*β* = 0.13 cm; SE = 0.32 cm; *p* = 0.69) or contralateral uninjured (*β* = 0.37 cm; SE = 0.34 cm; *p* = 0.28) limbs of previously injured participants. There were also no significant time*limb‐type interactions for the previously injured (*β* = 0.21 cm; SE = 0.22 cm; *p* = 0.34) or contralateral uninjured limbs (*β* = −0.02 cm; SE = 0.27 cm; *p* = 0.95) of previously injured participants, which suggests in‐season changes to BFlh fascicle length from baseline to follow‐up were comparable across all limb‐types (Figure 3).

### 3.2 Eccentric Knee Flexion Strength

**FIGURE 4:**
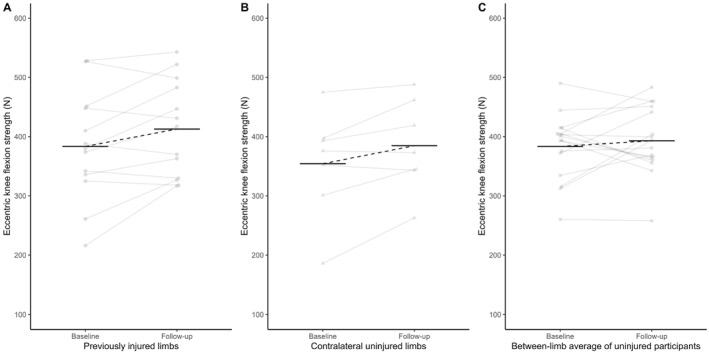
Eccentric knee flexion strength (*y*‐axis) at baseline and follow‐up (*x*‐axis) in previously injured limbs (A) and contralateral uninjured limbs (B) of previously injured participants, alongside the between‐limb average of uninjured participants (C). Individual data points and their change over time are shown in grey, while the horizontal black lines and dashed lines represent the average for each limb‐type.

The linear mixed‐effects model for eccentric knee flexion strength revealed no significant main effects of time (*β* = 9.53 N, SE = 12.97 N, *p* = 0.47), which indicates there was not an overall statistically significant increase in eccentric knee flexion strength from baseline to follow‐up. There were no significant baseline differences in eccentric knee flexion strength between uninjured participants and previously injured (*β* = −9.15 N; SE = 28.05 cm; *p* = 0.75) or contralateral uninjured (*β* = −12.14 N; SE = 29.78 N; *p* = 0.69) limbs of previously injured participants. There were also no significant time*limb‐type interactions for the previously injured (*β* = 18.29 N; SE = 19.22 N; *p* = 0.35) or contralateral uninjured limbs (*β* = 24.05 N; SE = 24.34 N; *p* = 0.33) of previously injured participants, which suggests in‐season changes to eccentric knee flexion strength from baseline to follow‐up were comparable across all limb‐types (Figure 4).

## 4 Discussion

To our knowledge, this is the first study to compare BFlh fascicle length and eccentric knee flexion strength changes between athletes with and without a previous hamstring injury following a standardised resistance training intervention. The main finding of this study was that irrespective of previous hamstring injury, following the 9‐week resistance training intervention including the NHE and staggered stance RDL once per week, BFlh fascicle length significantly increased, while eccentric knee flexion strength did not change significantly. These findings suggest that previous hamstring injury does not impact BFlh fascicle length or eccentric knee flexion strength changes following a relatively low‐volume, yet standardised resistance training intervention.

Our findings differ from a previous study, which found BFlh fascicle length improved across the first nine weeks of the season in previously uninjured elite Australian footballers, but in not those with a previous hamstring injury (Timmins et al. 2017). Unlike this previous study (Timmins et al. 2017), we standardised exposure to a 9‐week resistance training intervention across our cohort of 26 elite U20 male Gaelic footballers, which may explain why BFlh fascicle length improved significantly regardless of their hamstring injury history. Although statistically significant, these improvements in BFlh fascicle length were relatively small, with an average increase of 4% across all participants in our study, compared to average increases ranging from 7% to 24% following higher volume resistance training interventions (Behan et al. 2022; Carmichael et al. 2022; Crawford et al. 2025; Presland et al. 2018). In the context of acute hamstring injury rehabilitation, when resistance training is implemented at least twice per week, improvements in BFlh fascicle length have been shown to be greater in the injured limb compared to the contralateral uninjured limb, with average increases of 18% and 8%, respectively (Whiteley et al. 2022). Although BFlh fascicle length changes were of a similar magnitude between previously injured and contralateral uninjured limbs following weekly resistance training in our study, divergent changes may have been observed following higher volume resistance training interventions.

Our weekly resistance training intervention included the evidence‐based NHE (Medeiros et al. 2020; van Dyk et al. 2019), alongside the staggered‐stance RDL, which to our knowledge, has not been previously investigated. The NHE has been compared to the conventional bilateral‐stance and unilateral‐stance variations of the RDL, which elicit similar improvements in BFlh fascicle length (Crawford et al. 2025), and hamstring strength‐endurance (Behan et al. 2024), respectively. The conventional bilateral‐stance RDL has also been implemented concurrently alongside the NHE as a weekly resistance training intervention in elite under‐19‐year‐old soccer players (Lacome et al. 2020). Following this intervention, Lacome et al. found small improvements in BFlh fascicle length of 5% and a moderate increase in eccentric knee flexion strength of 11%, on average (Lacome et al. 2020). While these BFlh fascicle length improvements were similar to our study, we did not observe such changes in eccentric knee flexion strength (Lacome et al. 2020). Average eccentric knee flexion strength at baseline was greater in our participants (378 N) compared to those in the Lacome et al. study (325 N) (Lacome et al. 2020), which may have influenced their scope to get stronger (Ralston et al. 2017). Ultimately, our resistance training intervention was associated with small improvements in BFlh fascicle length, while maintaining eccentric knee flexion strength in elite U20 male Gaelic footballers with and without previous hamstring injury. In this context, further improvements in BFlh fascicle length and eccentric knee flexion strength may require a more intense or higher volume resistance training intervention, which warrants further scientific investigation.

The effect of resistance training volume on BFlh fascicle length and eccentric knee flexion strength has been investigated extensively in terms of isolated NHE interventions (Cuthbert et al. 2020). In the first of these investigations, similar improvements in BFlh fascicle length and eccentric knee flexion strength were observed following a low‐volume compared to high‐volume NHE intervention (Presland et al. 2018). However, the low‐volume intervention included an initial 2‐week period of exposure to two sets of four NHE repetitions twice per week (Presland et al. 2018), which differs from our weekly resistance training intervention. The most extensive investigation of BFlh fascicle length and eccentric knee flexion strength changes following four different training volumes, included a weekly prescription of two sets of four NHE repetitions (Behan et al. 2022), which we replicated in our study. Unlike our study, Behan et al. did not observe any significant changes in BFlh fascicle length, while eccentric knee flexion strength increased significantly following this low‐volume NHE intervention (Behan et al. 2022). These divergent findings could be explained by methodological differences between our study and Behan et al., including resistance training mode (concurrent NHE and staggered‐stance RDL vs. isolated NHE), intervention duration (nine weeks vs. six weeks), and participant population (elite U20 male Gaelic footballers vs. recreationally active men) (Behan et al. 2022).

In addition to the resistance training intervention, participants in our study were exposed to high‐speed running and sprinting as part of their regular on‐field training and matches during the first nine weeks of the season, which is typical of elite U20 Gaelic football (Mooney et al. 2021). Exposure to high‐speed running (i.e., at least 80% of maximum velocity) over an 8‐week period has previously been shown to explain 43% of the variance in BFlh fascicle length in elite rugby league players (McGrath et al. 2020). Therefore, the BFlh fascicle length adaptations observed in our study may not be solely attributed to exposure to the resistance training intervention and may have been influenced by this exposure to high‐speed running and sprinting. Nevertheless, our study provides ecologically valid evidence of BFlh fascicle length and eccentric knee flexion strength changes when a weekly resistance training intervention is implemented in addition to on‐field training and match demands during an elite U20 Gaelic football season.

We acknowledge that several methodological limitations must be considered when interpreting the findings of this study. Given the convenience approach to recruiting participants from single squad of elite U20 male Gaelic football players, the applicability of our findings beyond this context may be limited. In this context, we did not have access to medical imaging to confirm previous hamstring injuries, in terms of their specific anatomical location and severity of tissue damage. The lack of a non‐intervention control group in this study limits our ability to determine if BFlh fascicle length and eccentric knee flexion strength changes, or lack thereof, were a result of the resistance training intervention. Further to this point, it is beyond the scope of this study to decipher the relative utility of the NHE and staggered‐stance RDL in eliciting BFlh fascicle length and eccentric knee flexion strength changes. Finally, it is also unclear from our findings if additional resistance training volume or intensity would have elicited greater changes in BFlh fascicle length or eccentric hamstring strength, which should be explored in future studies.

## 5 Conclusion

In conclusion, our study shows that previous hamstring injury does not alter changes to BFlh fascicle length and eccentric knee flexion following a standardised exposure to a relatively low‐volume resistance training intervention in elite U20 male Gaelic footballers. These findings challenge the hypothesis that previous hamstring injury may blunt adaptations to training interventions. However, further research is needed to determine if higher volume resistance training interventions would result in divergent changes in BFlh fascicle length and/or eccentric knee flexion strength between athletes with and without previous hamstring injury.

## Funding

The authors have nothing to report.

## Ethics Statement

Confirmation of ethical compliance with the TUD Ethics Committee (reference no. REIC‐22‐223). All participants provided full informed written and oral consent before inclusion.

## Conflicts of Interest

The authors declare no conflicts of interest.

## Data Availability

The data that support the findings of this study are available on request from the corresponding author. The data are not publicly available due to privacy or ethical restrictions. Data was collected in the high‐performance facility of an elite‐level Gaelic football team.
